# How do health workers perceive and practice monitoring and evaluation of malaria control interventions in South-east Nigeria?

**DOI:** 10.1186/1472-6963-13-81

**Published:** 2013-03-06

**Authors:** Chinyere O Mbachu, Benjamin SC Uzochukwu, Obinna E Onwujekwe, Amobi L Ilika, Joseph Oranuba

**Affiliations:** 1 Health Policy Research Group, Department of Pharmacology and Therapeutics, College of Medicine, University of Nigeria, Enugu State, Nigeria; 2 Department of Community Medicine, University of Nigeria Teaching Hospital, Enugu State, Nigeria; 3 Department of Health Administration and Management, Faculty of Health Sciences, University of Nigeria, Enugu State, Nigeria; 4 Ministry of Health, Awka, Anambra State, Nigeria

**Keywords:** Monitoring and evaluation, M&E, Malaria, Knowledge, Perception, Practice, Health workers

## Abstract

**Background:**

The Anambra state Malaria Control Booster Project (ANMCBP) depends on an effective monitoring and evaluation (M&E) system to continuously improve the implementation of the malaria control interventions. However, it is not clear how the health workers that are expected to be the fulcrum of the malaria M&E perceive and practise M&E. The study was carried out to determine the knowledge, perception, and practice of Malaria M&E among selected health staff, and to identify related socio-demographic factors, including cadre of staff.

**Methods:**

A semi-structured questionnaire and an observation checklist were used to collect information from selected health workers in public primary health centres in all 21 local government areas of the State. Multistage sampling technique was used in selection of respondents. The questionnaire explored knowledge, perception and practice of malaria M&E from 213 health workers. The observation checklist was used to record the actual practice of malaria M&E as observed by trained supervisors.

**Results:**

Over 80% of health workers interviewed were able to correctly identify the malaria M&E forms; 25.4% knew the basis for categorizing Artemisinin-based combination therapy (ACT) into ACT1 - ACT4; 97.6% of the respondents felt there was need to keep proper records to have information available and 7.5% of them kept records because they were asked to do so. Over 90% of respondents reported correct practice of M&E, but on verification of actual practice, 55.6% of the respondents had at least one wrongly filled form, and half of these had none of their forms properly filled; about 68.4% of respondents had met specified timeline for data transmission. Differences observed in knowledge, perception and practice of M&E across age categories and cadres were only significant in ability to identify malaria M&E forms; diagnosis of malaria based on blood film microscopy result; perception of how age should be recorded; and reported practice of keeping data till they are requested. Among lower cadre of staff, gaps still exist in knowledge, perception and practice of malaria M&E.

**Conclusions:**

Gaps still exist in health workers’ understanding of malaria data management, perception of efficient data transmission and observed practice of malaria M&E.

## Background

Recognizing that there are proven and effective interventions against malaria, the Roll Back Malaria (RBM) Partnership was launched in 1998 by the World Health Organization (WHO), the World Bank, the United Nations Children’s Fund (UNICEF) and the United Nations Development Programme (UNDP), with the overall goal of halving the burden of malaria by 2010 [1]. Monitoring and Evaluation, (M&E), has been identified as a fundamental component of all health programs, and was adopted as one of the global strategies on which Roll Back Malaria anchors.

Policymakers and other stakeholders will often need to know whether a new policy or programme has been implemented in accordance with their expectations. Is the programme rollout progressing as planned, are the objectives being achieved, are the allocated funds being spent appropriately? Monitoring is the process of systematically collecting data to provide answers to such questions [2]. While monitoring tracks changes in program performance, evaluation determines the degree to which changes in health outcomes are the result of program activities. Program evaluations provide an empirical description of services provided, population served, and an assessment of whether the program delivered matches the conceptual framework on which it is based [3]. M&E, while improving the performance of health workers [4], often assumes that program and participants’ goals are mutually compatible [5]. Perceptions of M&E are framed by individual interests, and thus frequently fail to reflect the reality of M&E practice [6]. To evaluate a program, one has to be conscious of the stakeholders’ needs, problems, and perception [7].

The goal of RBM M&E system is to provide reliable information on progress in controlling malaria that can be used at local and national levels and can inform regional and global efforts [8]. It involves the collection of key data related to program objectives and operations, and analyses of these data to guide policy, programs and practiscs. Through a review of monitoring and evaluation capacity and practices in Africa, the RBM M&E Reference Group (MERG) reported that monitoring and evaluation within National Malaria Control Program has remained weak despite significant investment from RBM, and these weaknesses were primarily caused by limited human resources, lack of equipment, lack of an enabling environment and weak linkages with other programmes and partners [9].

**Figure 1:**
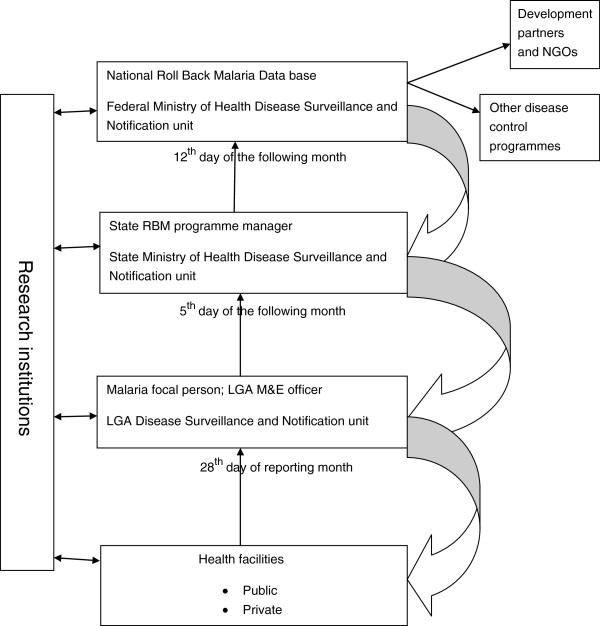
Flow chart for M&E data flow.

The M&E mechanism for the National Malaria Control Programme (NMCP) in Nigeria is designed to cover all control interventions such as prompt and effective case management including home-based care, integrated vector management (IVM) including use of long-lasting insecticide nets (LLINs), intermittent preventive treatment of malaria in pregnancy (IPTp) and communication for behaviour change [10]. Malaria surveillance is carried out within the confines of the National Health Management Information System (NHMIS) and in conjunction with the Epidemiology Division of the Department of Public Health, Federal Ministry of Health [10,11]. A monthly reporting system for malaria is in practice in Nigeria [11], data from all health facilities (public and private) are collected by the records unit of the health facilities using the standardized NHMIS tools and the modified NMCP M&E forms. They are collated, summarized and transmitted from the facility level to the National level (Figure 1).

The Anambra state Malaria Control Booster Programme (ANMCBP) depends on an effective M&E system so as to continuously improve the implementation of its interventions. However, it is not clear how the health workers that are expected to be the fulcrum of the malaria M&E perceive and practise M&E. This is especially pertinent following an extensive training of health staff on M&E and use of the modified NMCP M&E tools by the ANMCBP. An understanding of these issues will enable the refinement of M&E plans and modality for delivering the malaria control interventions for maximum impact. This study was conducted to determine the knowledge, perception, and practice of malaria M&E among health staff in selected primary health care facilities in Anambra state, Nigeria; and to identify related socio-demographic factors. The paper contributes to our understanding of the quality of M&E of malaria control programmes in Nigeria. This knowledge is useful for decision makers, especially malaria programme managers and those involved in health management information systems, in developing better M&E systems and recognising the potential influence of human factors in instituting good quality and efficient M&E systems.

## Methods

### Study area

The study was conducted in Anambra State, south-east Nigeria. Anambra State has a total of 21 Local Government Areas (LGA) and a population of 4,182,032 inhabitants whose major occupations are farming (75%), trading and fishing [12].

The State has 2 tertiary health institutions, one each owned and managed by the Federal Ministry of Health and the State Ministry of Health (SMOH); 34 secondary health facilities consisting of General, Comprehensive and Cottage Hospitals distributed across the whole LGAs and managed by the State Hospital Management Board of the SMOH; 382 Primary Health Care (PHC) centers and Health Posts which are managed by the LGAs with supervision from the Ministry of Health’s Department of Primary Health Care/Disease Control. The private sector also provides primary and secondary care services, most of which are in the urban areas [13]. The cadres of health workers employed by the public health facilities in the state are, in hierarchical order, doctors; midwives; nurses; nurse midwives; community health officers; community health extension workers; environmental health officers; and health attendants. However, primary health centres in the state are manned mostly by CHEWS, CHOs, and nurse-midwives in that order.

### Study design

The study was a cross-sectional study involving health workers in public primary health care facilities, who had received training on M&E for MCBP. All 21 LGAs in the State were included in the study. Data was collected from respondents using self and interviewer-administered questionnaires.

### Sampling and sample size determination

Adequate sample size was determined using a power of 80%, 95% confidence levels, error tolerated of 10% and health workers’ knowledge of Integrated disease surveillance and response (IDSR) reporting of 38% [14].

Multistage sampling technique was used to select respondents. Ten public primary health care facilities were selected using simple random sampling method from each of the 21 LGAs from a sample frame of health centres in the LGAs. A total of 210 health centres were selected, and one health worker who met the eligibility/inclusion criteria was selected from each of these facilities, making 210 respondents. The sample size was increased to 220 to make up for non response. The criteria for inclusion/eligibility were: health workers who have formal education and who had been trained on M&E tools for NMCP by any of the state’s malaria control project implementation facilitators.

### Data collection

Data was collected from respondents using semi-structured self and interviewer-administered questionnaires and by observation of completed M&E health facility forms by trained supervisors. Information was collected on their socio-demographic characteristics and on their knowledge, perception and practice of malaria M&E. The knowledge questions explored their understanding of the contents/components of the NMCP M&E tools (health facility forms) and their ability to properly identify and record a case of malaria in the forms. Health workers’ perception of malaria M&E was determined by assessing the way they value M&E and its importance in malaria control. In order to determine health workers’ practice of M&E, specific questions on how they fill out the forms and report stock-outs were asked, and all available completed malaria M&E forms were examined. An observation checklist was used to examine filled forms and it captured information on availability of the modified NMCP health facility forms (monthly summary and daily register forms); storage of the forms; cancellation of filled forms; number of forms not properly filled; up-to-date filling of M&E forms (capture of previous day’s malaria cases); timely sending of monthly health facility forms to LGA level.

### Data analysis

Data was entered and analysed using Epi info and SPSS version 17. Descriptive statistics was applied in the analysis of the socio-demographic characteristics of respondents. Open ended questions were first coded before analysis. Frequencies and proportions were calculated for categorical variables while means and standard deviations were calculated for non-categorical variables Correctness of knowledge and perception was measured by the ability to rightly answer the specific questions on each section. Correct practice was measured by ability to rightly answer questions on practice, and proper filling of completed forms that were observed. The relationship between some socio-demographic characteristics (age and cadre) and knowledge, perception and practice of M&E of the respondents was also determined. Chi square test was used for tests of significance for proportions of categorical variable, and all tests of significance were done based on a p-value of 0.05.

### Ethical approval

Ethical approval was obtained from the Health Research Ethics Committee of University of Nigeria Teaching Hospital, Enugu State, Nigeria and consent was sought from the LGAs’ authority.

Informed verbal consent was obtained from each respondent before the questionnaire was administered.

## Results

Data was collected from a total of 213 health workers, giving a response rate of 96.8%.

**Figure 2:**
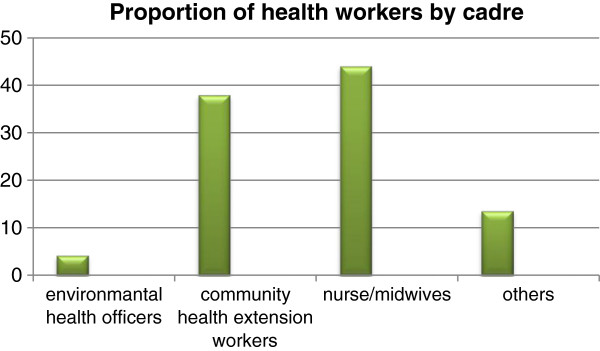
Proportion of health workers by cadre.

Socio-demographic characteristics of the health workers showed that the participants were within age group 24–59 years with mean age of 41.9 years (SD 7.0). Majority of the respondents, 111(52.3%), were within age group 40–49 years, and 203 (95.3%) of them were females. Figure 2 below represents proportion of respondents by cadre: 9(4.2%) were environmental health workers (EHOs), 81(38%) were community health extension workers (CHEWs), 94(44.1%) were nurses/midwives, and 29(13.8%) were others. Health workers included in the “others” category were community health officers, health attendants and assistants, and a doctor.

Results of health workers’ knowledge of malaria M&E shows that 173(81.5) and 175(82.3%) of them correctly identified the data collection forms; 187(87.9%) of them knew how to fill patients’ name; 145(68.1%) knew how to fill patients age; 131(61.5%) knew what a confirmed case of malaria is; 173(81.2%) knew what constituted severe malaria; and 54(25.4%) knew the basis for categorizing ACTs into four. (Table 1)

**Table 1:** Relationship between health workers’ age/cadre and knowledge of M&E of malaria control interventions

**Knowledge variables**		**Overall totals n(%)**	**Age categories**					**Cadres of health workers**				
			**20 – 29 (N=9)**	**30 – 39 (N=54)**	**40 – 49 (N=105)**	**50 – 59 (N=35)**	**x**^**2 **^**(p-value)**	**EHO (N=9)**	**CHEW (N=81)**	**Nurse/ midwife (N=94)**	**Others (N=29)**	**x**^**2 **^**(p-value)**
Identification of M&E forms	The health facility form 1 is called the ………… .	173 (81.5)	4 (44.4)	39 (72.2)	89 (84.8)	28 (80)	**10.20 (0.02)**	9 (100)	64 (79)	76 (80.8)	24 (82.8)	2.29 (0.52)
	The health facility form 2 is called the ………… .	175 (82.3)	8 (88.9)	35 (64.8)	84 (80)	28 (80)	**9.37 (0.03)**	7 (77.8)	67 (82.7)	74 (78.7)	26 (89.7)	1.77 (0.62)
What should be filled in forms	In filling the name of patient, which of the following is true?	187 (87.9)	7 (77.8)	46 (85.2)	92 (87.6)	30 (85.7)	0.01 (1.00)	9 (100)	69 (85.2)	84 (89.3)	26 (89.7)	0.84 (0.84)
	Which of the following is the best way to record the age of a patient/client?	145 (68.1)	0	0	3(2.9)	5(14.2)	**12.86(0.01)**	0	3(3.7)	4 (4.3)	2 (6.9)	0.95 (0.81)
	<2 years											
	Under which column is a blood film microcopy of +++ filled?	187(87.8)	5 (55.6)	50 (92.6)	89 (84.8)	27 (77.1)	**11.68 (0.01)**	8 (88.9)	70 (86.4)	82 (87.2)	27 (93.1)	0.93 (0.82)
Diagnosis of malaria	A confirmed case of malaria is one that ……………	131(61.5)	1 (11.1)	30 (55.6)	60 (57.1)	26 (74.3)	**11.50 (0.01)**	6 (66.7)	54 (66.6)	57 (60.6)	15 (51.7)	2.10 (0.55)
	Severe malaria refers to which of the following ……… .	173(81.2)	6 (66.7)	46 (85.2)	82 (78.1)	24 (68.6)	4.37 (0.22)	9 (100)	62 (76.5)	76 (80.8)	25 (86.2)	3.85 (0.28)
Malaria control interventions	The ACTs are classified as ACT1 – ACT4, based on ……… . .	54(25.4)	8 (88.9)	38 (70.3)	74 (70.5)	26 (74.3)	3.20 (0.36)	2 (22.2)	17 (21)	29 (30.9)	6 (20.6)	2.87 (0.41)

Health workers’ perception of M&E of malaria control interventions is presented in Table 2. It shows that 194(91.1%) of respondents think it is important to keep records of malaria and its control, and 208(97.7%) think that records should be kept so that information will be available. Some of the respondents, 16(7.5%), kept records because they were asked to do so. Most of the respondents, 155(72.8%), felt that both date of consultation and name of patient are important information to be documented from any patient visiting the health facility for treatment of malaria. It was found that 173(80.8%) 0f the respondents thought that age of patient should be recorded as age in numbers only (2 years), but 23(10.8%) of the respondents did not think any of the options for how to record age given was correct. On respondents’ perception of data transmission, 67 (31.5%) felt that effective data transmission should take into account the previous month’s updates from the field, 54 (25.4%) felt it should meet the specified timeline, 49 (23%) felt it should be done using the correct medium of transmission, and 119 (55.9%) felt it should comprise of all the above.

**Table 2:** Relationship between health workers’ age/cadre and their perception of M&E of malaria control interventions

**Perception variables, questions asked and responses**		**Overall totals n (%)**	**Age categories (years)**					**Cadres of health workers**				
			**20-29**	**30-39**	**40-49**	**50-59**	**x**^**2 **^**(p-value)**	**EHO**	**CHEWS**	**Nurse/midwife**	**others**	**x**^**2 **^**(p-value)**
Why do you think we need to keep proper records of all malaria cases seen at the health facility?	a) Because we are asked to do so	16 (7.5)	0	5(9.3)	5 (4.8)	3(8.6)	2.12(0.54)	1(11.1)	9(11.1)	5(5.3)	1(2.0)	3.10(0.38)
	b)To keep busy	2 (0.9)	0	0	0	1(2.9)	4.84(0.18)	0	0	2(2.1)	0	2.54(0.47)
	c)To have information on the number of malaria and other cases seen	208 (97.7)	9(100)	54 (100)	101(96.2)	34(97.1)	2.31(0.51)	9(100)	77(95.1)	93(98.9)	28(96.5)	1.75(0.63)
	d)Don’t know	1 (0.5)	0	0	0	1(2.9)	4.85(0.18)	0	0	1(1.1)	0	1.26(0.74)
Important information to be documented from a patient that visits a health facility include the following	a) Date of consultation	82 (38.5)	3 (33.3)	20(54.0)	36(34.3	16(45.7)	1.72(0.63)	5(13.5)	38(46.9)	28(29.8)	11(37.9)	6.16(0.10)
	b) Name of patient	91 (42.7)	3 (33.3)	22(40.7)	41(39.1)	18(51.4)	2.08(0.56)	5(13.50	42(52.0)	31(33.0)	12(41.4)	7.41(0.06)
	c) Place of work of patient	25 (11.7)	1(11.1)	5(9.3)	12(11.5)	6(17.2)	5.64(0.13)	0	12(14.8)	9(9.6)	4(13.8)	2.55(0.47)
	d) Only a and b	155 (72.8)	7(77.8)	42(77.8)	76(72.4)	19(54.3)	5.64(0.13)	6(66.7)	50(61.7)	76(80.9)	23(82.1)	8.20(0.04)
	e) Only a and c	12 (5.6)	0	3(5.6)	5(4.8)	3(8.6)	1.28(0.73)	0	5(6.2)	6(6.4)	1(3.4)	0.95(0.81)
Data transmission - For data transmission to be effective, it should ………	a) Take into account, the previous month’s updates from the field	67 (31.5)	2(2.2)	18(33.3)	31(29.5)	11(31.4)	0.25(0.97)	4(44.4)	23(28.4)	30(31.9)	10(34.5)	1.19(0.76)
	a) Meet the specified timeline	54 (25.4)	2(2.2)	19(35.1)	21(20	8(22.9)	3.61(0.31)	4(44.4)	25(30.9)	20(21.3)	6(20.7)	4.12(0.25)
	c) Be done using the correct medium of transmission	49 (23.0)	0	14(25.9)	20(1.9)	9(25.7)	3.75(0.29)	4(44.4)	18(22.2)	17(18.1)	11(37.9)	7.17(0.07)
	d) Have complete data/information	49 (23.0)	0	14(25.9)	22(2.1)	7(20)	2.96(0.40)	4(44.4)	18(22.2)	21(22.3)	6(20.7)	2.48(0.48)
	e) All of the above	119 (55.9)	4(44.4)	31(57.4)	60(57.1)	17(48.6)	0.74(0.86)	5(55.6)	42(51.9)	61(43.6)	13(44.8)	5.44(0.14)

Table 3 below presents health workers’ practice of M&E of malaria control interventions. Findings show that 200 (93.9%) of the respondents reported they kept malaria records with the health facility M&E forms, and 199 (93.4%) of them would summarize cases to get a total at the end of the month. On their practice of data transmission, 208 (97.7%) of them reported that they transmit malaria information to the LGA monthly, 204 (95.8%) reported that they sent it to the LGA focal person (they initiated the sending), and 151 (70.9%) 0f them send this information by hand.

**Table 3:** Relationship between health workers’ age/cadre and their practice of M&E of malaria control interventions

**Practice variables and questions asked**		**Overall totals n (%)**	**Age categories**					**Cadres of health workers**				
			**20-29**	**30-39**	**40-49**	**50-59**	**x**^**2 **^**(p-value)**	**EHO (N=9)**	**CHEW (N=81)**	**Nurse/ midwife (N=94)**	**Others (N=29)**	**x**^**2 **^**(p-value)**
Data collection	What do you use to keep records?											
	• Exercise book	21 (9.9)	1(11.1)	4(7.4)	10(9.5)	3(8.6)	0.27(0.97)	3 (33.3)	11(13.6)	6(6.4)	29 (100)	**9.42 (0.02)**
	• Any available paper	5 (2.3)	0	0	2(1.9)	2(5.7)	3.94(0.27)	1(11.1)	1(1.2)	2(2.1)	1(3.4)	3.57 (0.31)
	• Health facility M&E forms	200 (93.9)	7 (77.8)	53 (98.1)	95 (90.5)	33 (94.2)	3.89(0.27)	9 (100)	72 (88.8)	91 (96.8)	28 (96.6)	5.93 (0.12)
	• Any available form	6 (2.8)	0	1(1.9)	1(1.0)	3(8.6)	7.06(0.07)	0	1(1.2)	3(3.2)	2(6.9)	2.77 (0.43)
	What do you do with all the records you collect in a month?											
	• Put the records in my drawer	19 (8.9)	0	6(11.1)	7(6.7)	3(8.6)	1.82(0.61)	1(11.1)	9(11.2)	8(8.5)	1(3.4)	1.67 (0.64)
	• Summarize the cases to get a total	199 (93.4)	9 (100)	50 (92.6)	98 (93.3)	33 (94.2)	0.70(0.87)	9 (100)	75 (92.5)	87 (92.6)	28 (96.6)	1.32 (0.73)
	• Discard them, since the month has ended	2 (0.9)	0	0	1(1.0)	0	0.91(0.82)	0	0	2	0	2.54(0.47)
	• Take them to my house for safe keeping	0 (0.0)	0	0	0	0	-	0	0	0	0	-
**Data transmission** - At the end of the month when you have put together all the malaria cases, what do you do with the forms?	• Wait for the LGA malaria focal person to come for them	1 (0.5)	0	0	1(1.0)	0	0.91 (0.82)	0	0	0	1(3.4)	6.34(0.1)
	• Keep them with me till they are requested for	3 (1.4)	0	0	0	3(8.6)	**14.69 (0.002)**	0	1(1.2)	2(2.1)	0	0.90 (0.83)
	• Send it to the LGA malaria focal person	204 (95.8)	9 (100)	53 (98.1)	101 (96.2)	31 (88.5)	5.41 (0.14)	8 (88.9)	78 (96.2)	92 (97.9)	26 (89.7)	4.78 (0.19)
	• Don’t know	1 (0.5)	0	0	1(1.0)	0	0.91 (0.82)	0	0	0	1(3.4)	6.34(0.1)
How often do you send data on malaria and other cases to the LGA?	• Weekly	2 (0.9)	0	0	2(1.9)	0	1.84 (0.61)	0	1(1.2)	1(1.1)	0	1.46 (0.93)
	• Monthly	208 (97.7)	9 (100)	53 (98.1)	103 (98.1)	33 (85.7)	1.97 (0.58)	8 (88.9)	80 (98.8)	93 (98.9)	27 (93.1)	6.65 (0.08)
	• Once in three months	1 (0.5)	0	0	1(1.0)	0	0.91 (0.82)	0	0	0	1(3.7)	6.31(0.1)
	• Never	1 (0.5)	0	1(1.9)		00	2.80 (0.42)	0	1(1.2)	0	0	1.65 (0.65)
How do you send information on malaria data to the LGA	• Take the forms by hand to the LGA	151 (70.9)	6 (66.7)	41 (75.9)	71 (67.6)	23 (65.7)	1.73 (0.63)	8 (88.9)	59 (72.8)	60 (63.8)	24 (82.8)	5.75 (0.13)
	• Send anyone going to the LGA with the forms	16 (7.5)	1(11.1)	6(11.1)	5(47.6)	3(8.6)	2.66 (0.45)	0	6(7.5)	8(8.5)	2(6.9)	0.88 (0.83)
	• Put the forms in an envelope and send	47 (22.1)	2 (22.2)	7 (13.0)	26 (24.8)	10 (28.6)	3.74 (0.29)	0	15 (18.5)	28 (29.8)	4(4.3)	7.45 (0.06)

On observation to determine availability of malaria control interventions and M&E forms in facility, 111 (52.1%) of health facilities had any ACT in stock; 139 (65.3%) had LLINs in stock; 151 (70.9%) had SPs in stock; 3 (1.4%) had RDTs in stock; and 187 (87.8%) had M&E forms in stock.

On observation to verify actual practice of M&E, out of those who had M&E forms in stock (i.e. 187 health facilities), 83 (44.4%) had properly filled forms with no cancellations; 49 (26.2%) had cancellations in filled forms; half of those with improperly filled forms (52/104) had filled all observed forms wrongly; 114 (61%) of respondents that had M&E forms available had captured the previous day’s malaria cases in their forms, and 128 (68.4%) of them had sent the monthly forms to the LGA (i.e. met deadline for data transmission).

Tables 1, 2 and 3 also present relationships between some socio-demographic variables (age and cadre) and knowledge, perception and practice of M&E of malaria control interventions. Differences observed in knowledge, perception and practice of M&E across age categories were only significant in ability to identify malaria M&E forms; diagnosis of malaria based on blood film microscopy result; knowledge of what constitutes a case of severe malaria; perception of how age should be recorded; and reported practice of keeping data till they are requested (p < 0.05).

## Discussion

Previous experiences with M&E activities largely influence perceptions of M&E. A good proportion of our respondents knew how to identify the M&E forms, how to identify a case of malaria and what constitutes severe malaria. However, only about a quarter of them knew the basis for categorizing ACTs for treatment into four. This finding could suggest the role of health workers in stock out of ACTs for the most vulnerable group (children less than five years). Health workers in public facilities where ACTs are supplied through the AmFM programme have been known to give a combination of either four packets of ACT 1 or two packets of ACT 2 to adults. This practice depletes the stock for children by two or four for each adult treated.

M&E has been viewed as a highly sophisticated and technical tool used by senior staff for measurement, control and judgement of junior staff in organisations [6]. Though a small proportion of respondents in our study would keep records because they were asked to do so, majority of them felt it is important to keep records so information will be available. The level of perception of importance of record keeping and reason for doing so is an indication that most health workers may not perceive M&E as simply a tool for measurement and control, but also as a decision making tool. Perception of what constitutes effective data transmission was found to be relatively low and this could be explained by the limitations to communication which health workers in poor resource settings face, and this is expected to reflect on their actual practice of data transmission. Two major difficulties have been identified as responsible for the low perception of M&E in general, and they are: feelings of being controlled and perception of M&E tasks as an additional burden. The perception of M&E tasks as an additional burden is probably related to a poor understanding of the usefulness of M&E practice [15]. The current thinking is that a participatory approach to M&E will improve stakeholders attitude to M&E, and participation in development is generally accepted as a process that is fundamental to addressing issues of ownership and sustainability [16].

Knowledge of malaria M&E was found to significantly increase with increasing age. This could be attributed to the positive effect of work experience on knowledge and is in keeping with the study by Schmidt et al [17] which showed that job experience had a substantial direct impact on job knowledge and a smaller impact on performance capabilities. Knowledge was also found to increase with increasing staff cadre and this could be attributed to level of education. Higher levels of education have been associated with improved knowledge about the appropriate strategies for the prevention and treatment of malaria [18]. Perception variables were not found to follow any trend with regards to age, but there was relatively higher (though not significant) perception for age groups 30–39 and 40–49 years which may be accounted for by the fact that these fall into the management age group.

Discordance was found in the two methods that were used to assess the health workers’ practice of malaria M&E, whereas reported practice was found to be appropriate in over 90% of respondents in all but one of the questions asked, observation of actual practice was not as universal. Observation of filled forms showed that majority of them had improperly filled forms and half of these had filled all available forms wrongly. About two-thirds of the respondents had met specified deadlines for data transmission. This finding shows that actual practice is not always a direct result of knowing what to do, especially with respect to M&E. Knowledge, perception and practice gap was found among the lower cadres of health workers with perception and practice being higher than knowledge. In addition to receiving more supervision, these cadres of staff are the ones who are mostly targeted for training and retraining programmes, and though limited by knowledge and understanding still get to do what they are told to. In addition to training, good record keeping practices have been associated with positive attitude towards record keeping and duration of work [19].

The current gap between theory and practice can only be resolved it is argued by shifting the perspective from indicator- and data- driven M&E systems to learning-oriented systems [6,7,20,21]. Regular supervision of field staff data collection activities by the M&E officer(s) should have a supportive and formative orientation (i.e. aimed at providing field staff with the opportunity to consolidate and upgrade their relevant knowledge, skills and attitudes).

## Conclusions

Gaps still exist in health workers’ understanding of malaria data management, perception of efficient data transmission and practice of malaria M&E as observed by supervisors. Health workers’ knowledge, perception and practice of malaria M&E are not significantly affected by socio-demographic variable such as age and cadre.

In developing an M&E system, stakeholders’ empowerment is very important. Supervision of staff should in addition to being administrative, should fulfil educative and supportive functions as stated in the Kadushin’s model of supervision. In supportive supervision, the supervisor is able to create a learning environment and improve the work of his subordinates by being available and approachable, communicating confidence in the worker, providing perspective and opportunities for independent functioning and for probable success in task achievement [22].

## Competing interests

The authors declare that they have no competing interests.

## Authors’ contributions

OEO and BSCU conceived the study. All the authors participated in data collection and analysis. CM, BSCU and OEO drafted the manuscript. All the authors participated in revising the manuscript and agreed to the contents of the final version. All authors read and approved the final manuscript.

## Pre-publication history

The pre-publication history for this paper can be accessed here:

http://www.biomedcentral.com/1472-6963/13/81/prepub
